# Obesity and Its Clinical Implications in End-Stage Kidney Disease

**DOI:** 10.3390/medicina62010211

**Published:** 2026-01-20

**Authors:** Kristina Petruliene, Alanta Zilinskiene, Ruta Vaiciuniene, Kestutis Vaiciunas, Inga Arune Bumblyte, Egle Dalinkeviciene

**Affiliations:** 1 Department of Nephrology, Lithuanian University of Health Sciences, LT-50161 Kaunas, Lithuania; kristina.petruliene@lsmu.lt (K.P.); alanta.zilinskiene@lsmu.lt (A.Z.); ingaarune.bumblyte@lsmu.lt (I.A.B.); egle.dalinkeviciene@lsmu.lt (E.D.); 2 Department of Urology, Lithuanian University of Health Sciences, LT-50161 Kaunas, Lithuania; kestutis.vaiciunas@lsmu.lt

**Keywords:** obesity, end-stage kidney disease, obesity-related glomerulopathy, kidney transplantation

## Abstract

Both obesity and chronic kidney disease (CKD) are increasingly recognized as global epidemics. Their escalating incidence and far-reaching health implications highlight the urgent need for comprehensive prevention and management strategies. This review aims to clarify how obesity interacts with end-stage kidney disease (ESKD) and how to improve the management of obese patients receiving kidney replacement therapy. It also explores underlying mechanisms, current treatments, future directions, and ongoing controversies. By highlighting this intricate relationship, the review seeks to enhance clinical practice and promote further research toward more personalized care for this vulnerable population. Obesity is frequent in dialysis patients and creates challenges related to body composition, metabolism, and treatment. While higher body mass index (BMI) may appear to improve survival, this paradox does not offset the cardiovascular and functional risks of visceral and sarcopenic obesity. Obesity also increases post-transplant complications and can limit access to transplantation. Lifestyle changes rarely achieve lasting weight loss, whereas bariatric surgery—especially sleeve gastrectomy—can improve transplant eligibility with fewer complications. Weight-loss medications may be used before transplantation but remain insufficiently studied in ESKD. After transplantation, weight-reduction efforts should continue, with pharmacotherapy preferred over bariatric surgery. Comprehensive assessment strategies and individualized management approaches in ESKD patients are essential to optimize outcomes in this growing patient population.

## 1. Introduction

Obesity is a complex, chronic disease influenced by genetic, environmental, and social factors. It is a condition that can significantly worsen quality of life by reducing physical and mental health, as well as social well-being. Despite the preventive measures developed in recent years, the global prevalence and frequency of obesity continue to increase to such an extent that the World Health Organization considers it a global health epidemic [1]. In 2022, approximately 2.5 billion people were overweight, with 890 million suffering from obesity. Currently, one in eight people has obesity, indicating that obesity rates among adults have doubled and tripled among adolescents compared to 1990 [2]. It is predicted that in 2035, more than 1.5 billion people will live with obesity [3].

At the same time, the incidence of chronic kidney disease (CKD) is also rising—affecting more than 800 million people worldwide and becoming an increasingly significant cause of mortality [4]. CKD is a public health priority and a global issue, with projections suggesting that it will be one of the top five causes of death by 2040 [5]. As the global prevalence of CKD increases, more patients are reaching end-stage kidney disease (ESKD). The number of patients undergoing hemodialysis (HD), peritoneal dialysis (PD), or kidney transplantation is increasing. Simultaneously, with the obesity epidemic, more obese patients are requiring dialysis or a kidney transplant.

The relationship between obesity and kidney function is complex. On one hand, obesity is an established risk factor for the development and progression of CKD, leading to the ESKD in many cases. On the other hand, for patients already undergoing dialysis or after kidney transplantation, obesity presents additional challenges, complicating management and influencing outcomes. While obesity is often associated with increased morbidity and mortality in the general population, its effects on patients with kidney failure are more nuanced. Some previous studies have identified the “obesity paradox,” where a higher body mass index (BMI) and obesity may be associated with a potential protective effect and better survival in certain dialysis populations.

This review analyzes the complex relationship between obesity and ESKD, the mechanisms of obesity pathogenesis, clinical outcomes in patients undergoing HD, PD, and kidney transplant recipients. It also discusses various current and emerging obesity management strategies for patients on kidney replacement therapy. Managing obesity in ESKD is challenging. Approaches such as lifestyle changes, pharmacotherapy, and bariatric surgery differ depending on whether patients are on dialysis, preparing for kidney transplantation, or already living with a transplanted kidney. Due to the multifaceted relationship between obesity and ESKD, individualized, multidisciplinary treatment is essential.

The aim of this review is to deepen the understanding of the interaction between obesity and ESKD, to enable more effective management of obese patients undergoing kidney replacement therapy. In addition, it examines mechanistic insights to define current treatment approaches, future directions in the management of this condition, and existing controversies. By revealing the complex interplay between obesity and ESKD, this review seeks to contribute to the improvement of clinical practice and to encourage further research for tailored approaches to improve the quality of care for this vulnerable patient population.

## 2. Pathogenesis of Obesity-Related Chronic Kidney Disease

Obesity is an independent risk factor for the development of kidney disease, which could lead to structural and functional alterations, collectively referred to as obesity-related glomerulopathy (ORG) [6,7,8]. ORG diagnosis relies on two key components: clinical evidence of obesity (BMI of ≥30 kg/m^2^ in Western populations and ≥25 kg/m^2^ in Asian populations) and characteristic histopathological features on renal biopsy (glomerulomegaly, with or without evidence of focal segmental glomerulosclerosis (FSGS)) [9]. Approximately 10% of individuals with ORG may progress to end-stage kidney disease. Previous studies have documented serious adverse renal outcomes, including progression to ESKD or death related to chronic kidney disease, among individuals with a BMI ≥ 25 kg/m^2^, with the risk rising markedly when BMI exceeds 30 kg/m^2^ [10,11,12,13].

### 2.1. Inflammation

Recent studies emphasize that obesity-related inflammation is a critical contributor to chronic kidney disease pathogenesis. In obesity macrophages mostly produce pro-inflammatory cytokines and can change from anti-inflammatory macrophages (M2) to pro-inflammatory macrophages (M1) [14,15], resulting in a chronic inflammatory status. M2 release anti-inflammatory cytokines, such as interleukin-4 (IL-4), IL-10, and interleukin-1 receptor antagonist (IL-1Ra). M1 macrophages produce pro-inflammatory cytokines, such as IL-6, IL-8, IL-1β, IL-12, IL-23, and tumor necrosis factor alpha (TNFa) [15,16]. Increased activity of M1 macrophages and the effects of their secreted pro-inflammatory cytokines cause a background of chronic inflammation, with increased reactive oxygen species (ROS), as a signaling molecules for pro-inflammatory cytokines, production, and renal cell damage.

In a recent study it was established that macrophages with high expression of triggering receptor expressed on myeloid cells 2 (TREM2), found in the human adult kidney and in increased frequency in human diabetic and obese kidney tissue, may hold beneficial effects on worsening diabetic kidney disease, reducing inflammation in obesity-related kidney disease [17].

Despite macrophages effects in individuals with obesity-related CKD, white adipose tissue adipocytes secrete elevated levels of leptin, visfatin, and resistin, while adiponectin synthesis is diminished [17,18,19].

Elevated leptin contributes to inflammation, ROS generation, collagen deposition, and TGF-β1 activation [20]. Resistin promotes IL-1 and TNF-α expression and increases adhesion molecules such as VCAM-1 and ICAM-1 in the kidney. Visfatin (nicotinamide phosphoribosyltransferase) has been associated with higher VCAM-1, IL-6, TNF-α, and extracellular matrix production, partly via IKK/NF-κB and JAK2/STAT3 signaling pathways [21,22,23]. Collectively, these findings indicate that leptin, resistin, and visfatin drive inflammation, fibrosis, and CKD progression. In contrast, adiponectin, normally the most abundant adipocytokine, is reduced in obesity [24].

Adiponectin exerts anti-inflammatory effects primarily through NF-κB inhibition and promotion of macrophage polarization from the pro-inflammatory M1 to the anti-inflammatory M2 phenotype, thereby reducing pro-inflammatory cytokine production. Interestingly, serum adiponectin levels may be elevated in patients with obesity and CKD due to impaired renal clearance, with the highest concentrations observed in individuals with stage 5 CKD [25].

### 2.2. Oxidative Stress

Renal energy homeostasis largely depends on mitochondrial function. Impaired mitochondrial activity is the result of oxidative stress. High-fat diets induce changes in mitochondrial morphology and adversely affect mitochondrial biogenesis, dynamics, and quality-control mechanisms such as mitophagy [26].

ROS, which the accumulation of is promoted by the expansion of adipose tissue, are implicated in detrimental effects on intracellular organelles [27]. ROS induce lipid and protein oxidation, leading to cellular injury that predominantly affects glomerular and tubular structures, thereby contributing to proteinuria [28,29]. Consequently, the normal functions of the endoplasmic reticulum (ER), responsible for maintaining cellular homeostasis, and mitochondria, which play a central role in energy metabolism, become disrupted, resulting in renal dysfunction [30]. Elevated ROS levels can further provoke ER stress, leading to aberrant protein folding and apoptosis. Moreover, peroxisome proliferator-activated receptors (PPARs) significantly influence renal physiology through their roles in regulating oxidative stress and metabolic balance [31]. Evidence from animal studies indicates that mutations in PPAR-γ result in abnormal lipid accumulation and subsequent kidney impairment [32]. One of the cellular processes that protect the kidney is autophagy, an evolutionarily conserved mechanism that safeguards renal cells by removing damaged organelles and misfolded proteins [33]. By clearing ROS and excess lipids, autophagy maintains cellular homeostasis, and its deficiency exacerbates oxidative injury, underscoring its critical role in kidney health [34].

### 2.3. Insulin Resistance

Insulin resistance refers to a diminished responsiveness of insulin within its primary target tissues—liver, skeletal muscle, and adipose tissue—resulting clinically in impaired glucose tolerance, elevated blood glucose levels, and compensatory hyperinsulinemia. Adipocyte hypertrophy in obesity promotes the release of pro-inflammatory cytokines, free fatty acids, and leptin, ultimately contributing to the development of insulin resistance [35,36]. Hypertrophic adipocytes secrete TNF-α, which interferes with insulin action through increased phosphorylation of IRS-1 [37]. In contrast, TNF-α deficiency is linked to reduced circulating free fatty acids, which confers protection against insulin resistance [38,39].

Leptin normally regulates appetite and energy balance, but its chronic elevation induces leptin resistance, which paradoxically exacerbates insulin resistance. This effect is mediated in part by leptin’s ability to stimulate the production of pro-inflammatory cytokines, thereby amplifying inflammatory signaling and impairing insulin action [40,41].

### 2.4. Renin—Angiotensin—Aldosteron System (RAAS)

RAAS is a key hormonal and peptide-based pathway essential for regulating blood pressure, electrolyte levels, and overall fluid balance. It is initiated mainly in the kidneys, where reduced blood flow triggers the release of renin. Renin then cleaves angiotensinogen into angiotensin I, which is subsequently converted into angiotensin II by angiotensin-converting enzyme (ACE). Angiotensin II acts as a powerful vasoconstrictor and promotes the secretion of aldosterone from the adrenal cortex. In turn, aldosterone increases sodium reabsorption in the kidneys, helping to control fluid volume and stabilize blood pressure [42,43]. Excessive activation of the RAAS in obesity is a key contributor to the development and progression of ORG. This heightened activity is thought to result from several mechanisms, such as hemodynamic alterations caused by external compression of the renal parenchyma by intra-abdominal fat and increased intra-abdominal pressure, adipose tissue itself generating various RAAS components, and stimulation of the system through enhanced sympathetic nervous activity [44,45,46]. In obese individuals, systemic and local RAAS activity remains elevated even though salt and fluid retention would normally suppress renin release. This inappropriate activation is driven by the proinflammatory environment of adipose tissue, further promoting hypertension and metabolic disturbances [47,48].

Aldosterone—another major RAAS effector—is produced not only by the adrenal glands but also by adipose tissue, where local aldosterone synthase expression is increased in obesity. Elevated aldosterone levels in obese states promote insulin resistance and increase sodium retention, thereby worsening metabolic dysregulation. Through activation of mineralocorticoid receptors in adipocytes, aldosterone enhances adipogenesis and triggers inflammatory signaling pathways. This mineralcorticoid receptors-dependent signaling also stimulates key profibrotic mediators—particularly TGF-β and PAI-1—which are central to extracellular matrix accumulation and renal fibrosis in obesity-associated kidney disease [49,50].

### 2.5. Glomerular Hyperfiltration

In addition, obesity alters kidney blood flow by raising the glomerular filtration rate (GFR). This occurs because the afferent arteriole widens, allowing more plasma to reach the kidneys, which increases intraglomerular pressure, eGFR, and the filtration fraction (FF). Glomerular hyperfiltration (GH) is the key driving process behind kidney damage in ORG. Its crucial role in the progression of kidney disease was first recognized in diabetic nephropathy [51].

The elevated pressure within the glomerulus places mechanical strain on the filtration barrier, injuring podocytes. Over time, this leads to focal segmental glomerulosclerosis (FSGS) and thickening of the basement membrane, which contributes to enlargement of the glomeruli (glomerulomegaly) [51]. Histologically, GH is marked by enlarged glomerular and tubular structures and by areas where the glomerular basement membrane separates from podocytes [51,52]. Clinically, GH presents as increased albumin loss in the urine due to damage to the filtration barrier. Detecting albuminuria early is essential, before permanent nephropathy develops.

### 2.6. Microbiota Role

The gut microbiota includes the bacteria, viruses, fungi, and archaea living in the human intestines. Under healthy conditions, the host and gut microbiota remain in balance, but disruptions to this equilibrium—known as gut dysbiosis (characterized as increased ratio of the Gram positive Firmicutes to the Gram negative Bacteroidetes (F/B ratio)—are linked to numerous diseases, including CKD [53,54]. Obesity is now recognized as a condition marked by shifts in both the diversity and composition of the gut microbiota [55,56]. In many animal studies, the F/B ratio rises, though human findings are less consistent. Still, substantial evidence shows altered composition and reduced microbial diversity in obesity [57,58,59]. In obesity-related CKD, gut-derived uremic toxins appear to be central contributors. Levels of p-cresyl and indoxyl—converted in the liver to p-cresyl sulfate (PCS) and indoxyl sulfate (IS)—are elevated [60], along with other microbial products such as trimethylamine (TMA) and branched-chain fatty acids (BCFAs) [61].

### 2.7. Genetics

Genetic predisposition to general and central adiposity is a causal risk factor for developing CKD and kidney dysfunction. Key molecular mediators involved in obesity-related CKD include genetic polymorphisms in genes like AGT rs699, ACE I/D, LEP ENSSNP5824596, and FTO rs17817449. Alterations in these genes affect signaling pathways that accelerate renal damage by driving inflammation and fibrosis [62,63,64,65]. Adiposity genes influence CKD risk through both direct mechanisms and indirectly by mediating traditional risk factors like type 2 diabetes and hypertension [66,67].

### 2.8. Childhood Obesity and Kidney Involvement

Recent evidence suggests that childhood obesity represents a critical and independent risk factor for early kidney injury and the subsequent development of chronic kidney disease through complex and multifactorial mechanisms. Obesity affects renal hemodynamics and may promote hyperfiltration and other pathological changes from a very early age. These mechanisms are associated with insulin resistance and metabolic disturbances, as well as with adipokines—cytokines secreted by adipocytes—that can trigger adaptive or maladaptive responses in the kidney, ultimately contributing to the development of renal fibrosis. This supports the concept that the interaction between adipose tissue and the kidney constitutes a specific “adipose tissue–kidney axis,” in which neurohormonal, immunological, and inflammatory factors act in concert. These processes indicate that obesity-related kidney injury is not merely secondary to cardiometabolic diseases but may represent a direct, independent mechanism of renal dysfunction that begins in early childhood and persists into adulthood [68,69,70]. Early identification of at-risk children, together with targeted preventive and therapeutic interventions, is essential to mitigate long-term renal damage and reduce the burden of obesity-related kidney disease into adulthood.

**Figure 1:**
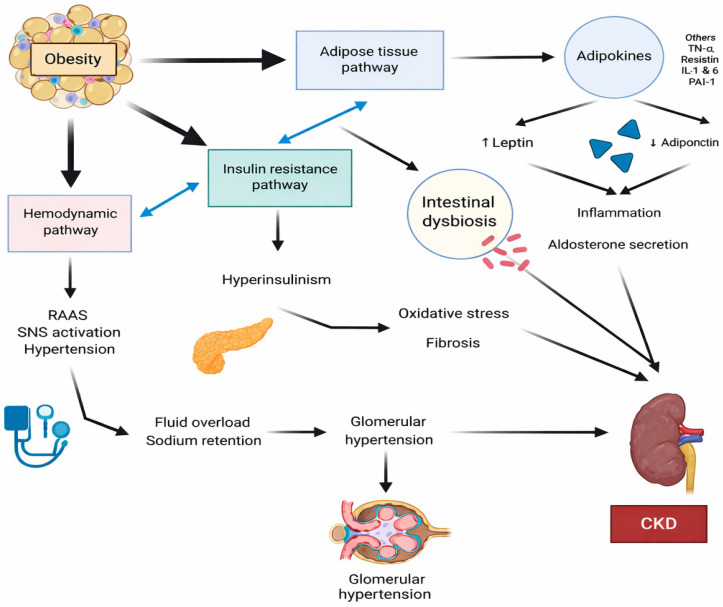
Pathways of kidney injury in obesity. Figure reproduced from: León-Román J et al. Obesity-Related Kidney Disease: A Growing Threat to Renal Health. Int J Mol Sci. 2025; 26:6641. © 2025 by the authors, licensed under CC BY 4.0 [71].

Pathways of kidney injury in obesity are shown in Figure 1 [71].

## 3. Obesity in Dialysis Patients

Obesity has emerged as a global epidemic affecting individuals across all age groups and clinical populations. Its growing prevalence is also seen in individuals with ESKD, adding challenges to the care of patients on dialysis [72]. Studies show that 30% to 45% of HD patients are living with obesity, with even higher rates documented in North America and the Middle East. Although the percentages are lower in European and Asian dialysis registries, they continue to rise as global patterns shift [73]. Kramer et al. investigated the increase in obesity in incident HD patients by age groups, finding a prevalence of obesity of 36% (95% confidence interval, 35–38%) and a prevalence of obesity of 44.6% (95% confidence interval, 43.0–46.2%) in persons with DM [74]. Many conditions commonly associated with obesity such as diabetes, high blood pressure, and metabolic syndrome are major causes of CKD, further increasing the number of obese patients entering dialysis programs. This is especially concerning because obesity can reduce eligibility for kidney transplantation, as many transplant centers use upper BMI limits when selecting candidates. Additionally, some healthcare providers consider obesity a relative or even absolute barrier to initiating PD, which may restrict treatment options available to patients with kidney failure [61,75,76]. In a retrospective, single-center study involving 1681 patients receiving PD, Than et al. reported that 37.7% of patients were overweight or obese at the initiation of PD. Notably, the prevalence of overweight and obesity increased over time, rising from 21.9% before 2000 to 47.3% in 2015 [77].

Obesity can make dialysis care more challenging. It may complicate the creation of vascular access and the placement of dialysis catheters. It is linked to higher rates of catheter issues and peritonitis in PD patients. People with obesity often need longer or more frequent HD sessions or extra PD exchanges to achieve adequate dialysis. Metabolic complications can be more significant, particularly with PD, and excess weight may reduce a patient’s suitability for kidney transplantation [78]. So, the growing rate of obesity creates additional difficulties in obtaining, providing, and sustaining high-quality care for people with CKD, including those receiving HD, PD, or a kidney transplant [78].

### 3.1. Assessment of Obesity in Dialysis Patients

The pattern of fat accumulation in CKD patients is different from that seen in the general population. These patients often have a disproportionate amount of visceral fat and sarcopenic obesity, characterized by loss of muscle mass combined with excess fat. This is especially common in this group of patients due to factors such as inflammation, anabolic resistance, metabolic acidosis, and multiple comorbid conditions [72]. Such a body composition pattern is linked to higher rates of frailty, hospital admissions, and mortality. Although BMI is still widely used, it can be misleading in CKD because fluid overload frequently affects weight. The distribution of body fat across different compartments might be a more accurate indicator of obesity in this group of patients. Tools like waist circumference and waist–hip ratio are often used to estimate visceral fat, which has a strong association with poor clinical outcomes [73]. Waist circumference is strongly linked to both overall and cardiovascular mortality, regardless of BMI adjustment. It is a simple, practical measurement that can serve as an indicator of abdominal fat. Other assessment methods include bioimpedance spectroscopy, which helps to estimate fat and lean tissue, and DEXA scanning—considered the gold standard for evaluating body composition in CKD. Functional measures like handgrip strength can also be used to detect sarcopenia. Using these approaches together enhances risk assessment and supports better clinical decision-making [79].

### 3.2. Clinical Consequences of Obesity in Dialysis Patients

#### 3.2.1. The “Obesity Paradox” in Dialysis Patients

In the general population, obesity is widely recognized as a major driver of metabolic diseases, including type 2 diabetes and non-alcoholic fatty liver disease, as well as a range of cardiovascular disorders. It has also been linked to conditions such as Alzheimer’s disease, depression, osteoarthritis, obstructive sleep apnea, gonadal dysfunction, gastroesophageal reflux, and several forms of cancer [73]. Obesity is further known to contribute to both the onset and progression of CKD [80]. However, among individuals undergoing dialysis, these relationships are less straightforward because of overlapping risk factors and the physiologic effects of uremia. Some studies report that obesity in dialysis patients is associated with increased left ventricular hypertrophy and more severe hypertension, whereas other investigations demonstrate no clear influence or even a paradoxical survival benefit associated with higher BMI [77]. A review of US Renal Data System records that included 418,055 HD patients showed that higher BMI was linked to improved survival, even among individuals with very high BMI values [80]. In this analysis, elevated BMI was also associated with fewer hospital admissions and reduced mortality across all categories. Likewise, a meta-analysis of 22 studies involving HD populations demonstrated a linear relationship between BMI and survival. Each 1 kg/m^2^ increase in BMI decreased the risk of all-cause mortality by 3% and cardiovascular mortality by 4% [81,82]. Kalantar-Zadeh introduced the concept of “reverse epidemiology of obesity” to describe this unexpected observation that higher body weight may be linked to better survival outcomes in dialysis patients [83]. However, most studies exploring the relationship between obesity, CKD, and mortality are observational and have relatively short follow-up periods. Long-term evidence in HD patients is limited. Data from PD populations are even more sparse, and findings remain contradictory. In a large cohort of 10,896 PD patients, researchers examined how baseline serum creatinine, a marker of muscle mass, and its change over the first three months related to overall mortality. Using patients with serum creatinine levels of 8.0 to <10 mg/dL as the reference group, those with levels <4.0 mg/dL and 4.0 to <6 mg/dL had 36% and 19% higher mortality risks, respectively. In contrast, patients with levels of 10.0 to <12 mg/dL, 12.0 to <14 mg/dL, and >14 mg/dL showed 12%, 29%, and 36% lower mortality risks. A drop in serum creatinine of more than 1.0 mg/dL during the first three months was also linked to an additional increase in the risk of death [84]. More recent findings suggest that frailty may modify the effect of obesity on outcomes in dialysis patients. In a study of 267 Chinese PD patients, Chan et al. found that frail patients had a higher waist–hip ratio, indicating greater central adiposity, but not a higher BMI. Among non-frail individuals, those with a higher waist–hip ratio had significantly better two-year cardiovascular survival (91.3% vs. 74.4%) and fewer cardiovascular-related hospital admissions. However, these associations were absent in frail patients. The findings suggest that central obesity may provide a survival advantage in non-frail PD patients, but not in those who are frail [85].

Multiple explanations have been proposed for the so-called “obesity paradox.” However, it remains uncertain whether the relationship between higher BMI and improved survival represents a true causal effect [79]. Several hypotheses have been proposed to support a biologically plausible explanation for the obesity paradox in CKD, suggesting protective factors like higher nutritional reserves, a more favorable profile of circulating lipoproteins and sequestration of inflammatory cytokines within adipose tissue, a more stable blood pressure and circulation during dialysis, increased levels of circulating TNF-α receptors, and less activation of stress-related hormones. Patients with lower BMI may produce proportionally more uremic toxins, while fat tissue in patients with higher BMI could act as a reservoir that slows the movement of fat-soluble uremic toxins through the body—an effect that would not occur in individuals with little body fat [86]. The main theory focuses on protein-energy wasting (PEW), which is common in people with advanced CKD. PEW develops through inflammatory pathways, where increased levels of cytokines like IL-6 and TNF-α suppress appetite, promote muscle breakdown, and result in low albumin levels. The loss of fat and muscle mass, together with ongoing inflammation, raises the risk of cardiovascular disease and death by damaging the vascular endothelium. Obesity may lessen the impact of PEW and its consequences. People with more body fat are less likely to experience PEW during times of poor nutrition or inflammation because they have larger energy and protein stores to draw from. Conversely, patients with limited nutritional reserves may be more susceptible to inflammatory damage [79]. However, the strength of these associations is questioned when considering that BMI does not differentiate between fat and muscle mass, does not distinguish central from peripheral fat distribution, and cannot separate visceral from subcutaneous adiposity—each of which has different associations with inflammation, metabolic syndrome, cardiovascular disease, malignancy, and other adverse outcomes in the general population [87]. Supporting this limitation, a study of more than 70,000 HD patients reported that the lower mortality risk associated with higher BMI was present only among individuals with increased muscle mass and not among those with high fat mass. Waist circumference, a marker of central adiposity, showed a direct association with cardiovascular and all-cause mortality, independent of BMI [87]. The evidence suggesting that obesity provides clinical benefit in dialysis patients is far from conclusive, and there is no justification to recommend that patients starting dialysis attempt to increase their weight to reach an obese range. On the contrary, patients with ESKD and obesity should be encouraged to lose excess weight because obesity introduces multiple practical and clinical challenges in their care. Crucially, reliance on BMI alone is insufficient—systematic assessment of sarcopenia and visceral adiposity is essential to accurately evaluate nutritional status, stratify risk, and guide individualized management. There is a need to implement standardized measures of sarcopenia and visceral adiposity in routine CKD clinical practice. Prognosis can improve when a patient reduces weight sufficiently to become eligible for kidney transplantation. Moreover, weight loss in obese patients who are already listed for transplantation may reduce the risk of perioperative and postoperative complications [76].

#### 3.2.2. Dialysis Modality Considerations for Obese Patients

The best dialysis method for patients with obesity remains uncertain, because excess body weight creates several difficulties for both HD and PD, including establishing access, delivering sufficient dialysis, and managing practical aspects of treatment [76]. Although patients with obesity undergoing PD have a higher risk of complications compared with non-obese PD patients, their overall survival is comparable to that of patients treated with HD, suggesting that obesity should not be considered as a contraindication to PD [88,89]. Dialysis modality selection should therefore be guided by a shared decision-making process that carefully balances risks and benefits while incorporating the patient’s clinical history, individual risk profile, and personal preferences. With appropriate technical and clinical adaptations—such as meticulous exit-site selection, modified catheter insertion techniques, use of icodextrin for long dwell exchanges, and implementation of incremental PD to reduce glucose exposure—PD can be safely and effectively delivered in patients with obesity [90].

#### 3.2.3. Complications of Obesity in HD Patients

Obesity can interfere with the assessment of dialysis adequacy, since Kt/V calculations may underestimate the dialysis dose required for larger patients. In addition, excess adipose tissue can make catheter insertion, arteriovenous fistula (AVF) maturation, and cannulation more difficult. Although most studies suggest that primary patency rates of AVF and arteriovenous grafts (AVG) are similar in obese and non-obese patients, long-term access survival may be reduced in patients with severe obesity [91]. The shorter durability of vascular access in patients with obesity may be attributable to compression of the outflow vein by increased subcutaneous adipose tissue. In addition, greater access depth within the subcutaneous tissue can make cannulation technically challenging. Adjunctive interventions, such as lipectomy, access superficialization and liposuction, have been shown to facilitate successful cannulation and improve overall vascular access patency and survival [91,92]. Central venous catheter placement is more challenging in patients with obesity because conventional anatomic landmarks may be obscured, even when ultrasound guidance is used [93]. Caring for individuals with obesity often requires additional resources. To reach target Kt/V values, dialysis time or session frequency may need to be increased, which is not always practical due to dialysis unit constraints or patient refusal [94,95]. Patients with obesity undergoing HD who have limited mobility may require additional resources, including extra-large wheelchairs, stretchers, increased staffing to assist with patient transfers, and specialized equipment such as oversized blood pressure cuffs and bariatric dialysis chairs [96]. Patients with obesity have a higher prevalence of comorbid conditions, including obstructive sleep apnea, pulmonary hypertension, and heart failure, which are associated with an increased risk of intradialytic hypotension and difficulty in achieving dry weight [92]. Obesity is a well-established risk factor for proximal calciphylaxis and is thought to contribute through expansion of adipose tissue, which increases tensile stress on calcified arterioles and further compromises local blood flow. Necrotic lesions frequently involve extensive areas of skin and subcutaneous tissue, thereby markedly increasing the risk of secondary infection. Consequently, aggressive and comprehensive wound care is critical for patient survival [78].

#### 3.2.4. Complications of Obesity in PD Patients

Older publications suggested that obesity was a barrier to PD, based on the belief that it would not provide sufficient clearance. However, with the development of automated cycling PD and increased experience with PD overall, this belief has been disproven. Multiple studies now show that obese patients can successfully receive PD, including continuous ambulatory PD. This is particularly true for patients who still have remaining kidney function, which tends to be preserved longer with PD than with HD [97,98]. Although obesity is not a contraindication to PD, patients with obesity experience higher rates of complications, including catheter malfunction, exit-site infections, peritonitis, hernias, and dialysate leaks. Increased visceral adiposity is associated with greater omental volume, which may envelop the PD catheter, obstruct drainage ports, or displace the catheter from the pelvis, thereby contributing to catheter dysfunction. Evidence regarding the benefit of prophylactic omentectomy or omentopexy at the time of catheter placement remains inconsistent. In one study, prophylactic omentectomy was associated with a catheter malfunction rate of approximately 2%, compared with historical rates exceeding 2–10% [99]. The risk of exit-site infections and peritonitis is increased due to challenges in exit-site care and a heightened susceptibility to skin and soft tissue infections [100,101]. PD-related infections remain the leading cause of transfer to HD. In patients with increased subcutaneous adiposity, pericatheter leakage and impaired wound healing represent important risk factors for exit-site and tunnel infections. Preventive strategies include placement of a readily visible exit site to facilitate routine care, prevention of constipation, and strict adherence to hand hygiene—all of which are essential to reducing infectious complications [102]. Positioning the catheter exit site above the beltline avoids skin folds where moisture may accumulate and improves patient visibility of the exit site, thereby facilitating effective exit-site care. Presternal catheters may be considered in selected patients; however, their increased length is associated with longer fill and drain times, a higher risk of kinking or obstruction, and an increased likelihood of peritoneal–pleural leaks compared with abdominally placed PD catheters [90]. Patients with obesity may be at increased risk of metabolic complications during PD. Use of icodextrin has been associated with less weight gain and allows reduced reliance on hypertonic glucose-based dialysate solutions [103]. Achieving adequate solute clearance in patients with obesity may necessitate longer treatment durations or more frequent exchanges. Obesity is associated with elevated intra-abdominal pressure, which is further increased by dialysate instillation and thereby heightens the risk of hernias and dialysate leaks. With recognition of the specific challenges related to PD catheter placement, meticulous attention to exit-site care, proactive management of metabolic complications, incorporation of incremental PD, and individualized prescriptions that balance practicality with adequate solute and volume control, patients with obesity can achieve favorable outcomes on PD [78,99,104]. Glucose absorption associated with PD has raised concerns regarding additional weight gain in dialysis patients. Earlier studies estimated that approximately 100–300 g of glucose were absorbed daily from glucose-containing dialysate. However, with current PD practices—including widespread use of automated cyclers with shorter dwell times and the use of dialysate bags with varying glucose concentrations, as well as icodextrin for long dwell exchanges—overall glucose absorption is substantially lower [105]. Nonetheless, glucose absorption during PD may contribute to additional caloric intake and potential weight gain. However, weight gain is also commonly observed in patients undergoing HD. This phenomenon may reflect an initial improvement in appetite and nutritional status following the removal of uremic toxins with either modality. In PD, the additional caloric load from dialysate glucose may promote early satiety, potentially leading to a compensatory reduction in oral dietary intake [106,107].

Evidence on impact of obesity in dialysis patients is summarized in Table 1.

## 4. Obesity and Kidney Transplantation

### 4.1. Obesity in Candidates to Kidney Transplantation

Obesity is associated with cardiovascular, metabolic, and surgical complications after kidney transplantation: delayed wound healing, infections, hernias, lymphoceles, and thrombotic events [109]. There are data that obesity is a risk factor for graft loss and death, as well as biopsy-proven acute rejection [110].

Meta-analysis of 26 studies showed increased mortality (RR = 1.52), delayed graft function (RR = 1.52), acute rejection (RR = 1.17), wound infection ant dehiscence (RR = 3.13 and 4.85), new onset diabetes after renal transplantation (NODAT) (RR = 2.24), and length of hospital stay (2.31 days) in kidney transplant patients with BMI > 30 kg/m^2^. However, patient survival expressed in hazard ratios was in significant favor of high BMI recipients [111]. Two other meta-analyses found that obesity was associated with higher mortality in kidney transplant recipients [112,113]. In the meta-analysis, which included 55 studies, 15,458 kidney transplantation cases, BMI was an independent risk factor of NODAT (mean difference, 1.88; 95% CI, 1.48–2.27) [113]. Overall, graft loss and death were associated with obesity only in the analysis of studies of kidney transplants performed before year 2000, and no association of obesity with graft loss and death was found in the analysis of studies that evaluated kidney transplants performed after the year 2000 [114,115].

Although results of kidney transplantation (KT) are worse in obese patients, kidney transplantation in this patient group provide survival benefits when compared with remaining on dialysis [116]. There is no consensus between different transplant centers in BMI threshold for listing patients on transplant waiting list [117,118], and obesity can be a barrier to transplant access. Studies using United Network for Organ Sharing (UNOS) data show that 20% of transplant centers did not list patients with BMI > 40 kg/m^2^ because of worse outcomes, more difficult operation, and increased treatment costs [119]. Six surveys from Europe, the United States, and the UK reported extreme obesity being a potential contraindication for kidney transplantation. The BMI thresholds for kidney transplantation ranged from 30 to 34 kg/m^2^ (2–24% of surveyed centers), 35–39 kg/m^2^ (12–51% of surveyed centers), to >40 kg/m^2^ (6–62% of surveyed centers) [120,121,122].

DESCARTES (Developing Education Science and Care for Renal Transplantation in European States) Practical Guidelines on Management of Obesity in Kidney Transplant Candidates recommend accepting people with ESKD and a BMI of 30–34 kg/m^2^ for kidney transplantation [123]. The Kidney Disease: Improving Global Outcomes (KDIGO) Clinical Practice Guideline on the Evaluation and Management of Candidates for Kidney Transplantation 2020 confirms that BMI > 40 kg/m^2^ is associated with the increased risk of post-operative complications but suggest not to exclude patients from transplantation only because of obesity [124].

Various weight loss possibilities (behavioral, nutritional consultations, pharmacotherapy, bariatric surgery) help obese ESKD patients to overcome a barrier for kidney transplantation [125].

Before administration of pharmacotherapy or surgery, there is high importance for obese ESKD patients to change their lifestyle and to increase a physical activity (at least 150 min of moderate intensity physical activity per week) [126,127]. Unfortunately, lifestyle modifications, in particular dietary counseling, rarely lead to sustained weight loss. Increasing physical activity is also rarely effective in the long run [128]. Patients with ESKD face challenges represented by renal-specific diet restrictions and limited exercise capacity (highly prevalent comorbidities, anemia, and post-dialysis fatigue) [104]. In this situation, bariatric surgery may help to achieve weight loss more rapidly, with over 50% of total weight loss occurring within the first 2 months [129]. Clinical studies show that metabolic bariatric surgery may be more effective in achieving kidney transplant listing (shorter time to waitlisting and a higher likelihood of receiving kidney transplantation) than the nonsurgical approach [130,131].

Bariatric procedures can be performed either open or laparoscopically. There are three types of bariatric surgery:

1. Bariatric surgery, which reduces the size of the stomach (intra-gastric balloon placement, adjustable laparoscopic gastric banding, and laparoscopic sleeve gastrectomy (LSG)).
2. Bypass a segment of small intestine to cause malabsorption (biliopancreatic diversion with and without duodenal pouch).
3. Combination of reduction in the stomach size and bypass of the proximal intestine with a gastrojejunostomy (Rouxen Y gastric bypass (RYGB)).

The most popular are laparoscopic sleeve gastrectomy (LSG) and laparoscopic Roux-en-Y gastric bypass (RYGB). Both procedures have been proven to be effective for significant weight loss in ESKD patients (up to 80% within 24 months) providing the possibility to be listed for kidney transplantation [132]. Metanalysis of 19 studies (288 patients) showed that during 32.9 ± 21.4 months patients lost an average of 11 kg/m^2^ (mean BMI decreased from 43.9 ± 5.3 kg/m^2^ to 33.7 ± 5.4 kg/m^2^ (*p* = 0.003)), 50.3% were listed, and 29.5% were transplanted during 19.9 + 14.3 months after bariatric surgery [133]. In a study where a Markov decision analytic model was used to evaluate best option for ESKD patients to reach a BMI < 35 kg/m^2^, the medical method for weight loss was compared with bariatric surgery (sleeve gastrectomy (SG) and Roux-en-y gastric bypass (RYGB)). RYGB patients had a higher chance of transplantation and improved long-term survival: RYGB provided 1.3 additional years of life compared with SG and 2.6 additional years of life compared with medical weight loss [134]. Another study created a decision analytic Markov state transition model to evaluate the effectiveness of sleeve gastrectomy, glucagon-like peptide-1 receptor agonist (GLP-1 RA), and dual GLP-1/GIP RA Tirzepatide, a dual GLP-1 RA and glucose-dependent insulinotropic polypeptide receptor agonist (GIP RA) in morbidly obese ESKD patients. At 5 years, highest efficacy was predicted for SG (14.74% patients receiving a transplant), less effective being the use of GLP-1/GIP RA (9.06% of patients receiving KT), and the least efficacy was predicted for the use of GLP-1 RA (4.83% KT) [135].

Although a bariatric surgery is reported to be effective for weight loss before kidney transplantation, there is no consensus on the best time and best operation. However, sleeve gastrectomy is associated with less complications [133]. DESCARTES guidelines suggest laparoscopic sleeve gastrectomy as a preferred option of bariatric surgery in KT candidates [123]. Compared to Roux-en-Y gastric bypass, SG is associated with less surgical complications, no risk of kidney stones or oxalate nephropathy, and less problems with medication absorption [131].

Another solution to avoid early postoperative complications, especially wound complications of morbidly obese ESKD patients, is a robotic kidney transplantation, which is increasingly used in recent years [136,137]. A 10-year single center study with 248 obese ESKD patients after robotic-assisted kidney transplantation (RAKT) concluded a good 3-year graft and patient survival and minimal risk of side effects. Moderate increase in a warm ischemia time correlated positively with BMI and DGF (11%) [137]. Another group from the US reported on the safety and efficacy of combining robotic-assisted sleeve gastrectomy and robotic KT in obese (class II and III obesity) KT recipients. Between 2012 and 2019, 20 renal transplant recipients participated in a randomized controlled trial comparing the safety and efficacy of robotic sleeve gastrectomy (RSG) with RAKT compared with RAKT alone in obese ESKD patients. The combination of RSG and RAKT improved BMI and had similar graft function compared with RAKT alone [110,138].

The possibility to use a pharmacotherapy for a weight loss is commonly explored in the general population but less studied in ESRD and renal transplant patients. Incretin mimetics, including GLP-1 RAs and GIP RA, suppress appetite and slow gastric emptying [139]. There are insufficient data on the use of GLP-1 Ras and GLP-1/GIP RA in advanced CKD. ESKD patients and patients after kidney transplantation were excluded from large randomized controlled trials (RCTs) of GLP-1RA in type 2 diabetes mellitus (DM) and obesity. In small RCTs liraglutide reduced weight by 1–2 kg of patients with type 2 diabetes on dialysis, compared with placebo. In prospective studies with inclusion of ESKD, patients using semaglutide were able to reduce weight up to 8 kg over 12 months [140].

A single-center retrospective study evaluated 36 patients on dialysis who were prescribed semaglutide (GLP-1 RA). Of patients who were ineligible for transplant due to elevated BMI, 48.2% achieved waitlist activation after successful weight loss. Treatment-limiting gastrointestinal side effects occurred in 16.7% of patients [141]. In a prospective 12-week, open-label trial with 13 dialysis patients with BMI ≥ 30 kg/m^2^, semaglutide was given as a treatment for weight reduction before kidney transplantation, and the weight reduction was 4.6 ± 2.4 kg (ranged from 2.0 to 9.7 kg). One patient discontinued treatment due to nausea/vomiting, and six patients reported side effects [142].

Despite all the described ways for obese ESKD patients to be transplanted, there are no prospective studies which would compare all the possibilities and evaluate a longer time on dialysis while seeking weight reduction [110]. A prospective cohort study with 919 KT recipients showed that only intentional weight loss before KT is beneficial to ESKD patients, while unintentional weight loss increased a risk of graft loss and mortality after KT [143]. There are considerations that BMI alone should not be a contraindication for kidney transplantation. We also have to account for advanced age (>65 y) with poor functional status, calcifications of the vessels, cardiovascular status, hypotension, and pulmonary hypertension [110,143].

### 4.2. Obesity After Kidney Transplantation

Weight gain is also a common problem after kidney transplantation, especially during the first year. The prevalence of obesity is 20% among these patients. The most common causes are end of dietary restrictions, increased appetite, lack of physical activity, and immunosuppression (corticosteroids and calcineurin inhibitors) may increase hyperglycemia [76].

Weight loss, renin-angiotensin system blockade and SGLT2i are recommended for these patients’ [76]. A healthy diet and exercise remain the first-line treatment for obesity [109]. But, a high rate of non-compliance with recommendations of a diet and physical activity is reported in kidney transplant patients.

A single-blind, small-size (36 participants) randomized controlled trial (the INTENT trial) was conducted to determine whether an intensive nutrition and exercise intervention would reduce post-transplant weight gain. After patients’ randomization to intensive nutrition intervention (12 dietitian visits; 3 exercise physiologist visits over 12 months) or to standard nutrition care (4 dietitian visits), there were no differences in both groups [144]. Another pilot single-center RCT tested the effectiveness of a cognitive-behavioral and nutritional counseling for weight reduction following KT versus control group. A total of 56 kidney transplant recipients were included. The main outcome—a 5% weight loss—was not achieved in intervention group. Possible reason—COVID-19 pandemic interrupting the study with an influence of health behaviors [145].

Talking about pharmacotherapy for weight reduction, incretin-based medications are preferred. For patients with type 2 diabetes, tirzepatide was more effective for weight reduction and control of glycated hemoglobin after 40 weeks of treatment as compared with semaglutide [146]. However, semaglutide was also shown to be effective on weight loss and HbA1C reduction, with no negative effects on transplant function in KT recipients [147]. But, the incretin-based medications may worsen gastrointestinal side-effects, which are common for immunosuppressive medications, especially mycophenolate mofetil. There are no RCTs with GLP1-RAs in kidney transplant patients [148]. Eight observational studies in kidney transplant patients reported a body weight loss (2–5 kg) in patients using GLP1-RAs with inconsistent effects on eGFR [149].

Bariatric surgery after KT is effective for significant weight loss with associated reduction in comorbidities. But, this benefit is associated with delayed wound healing, increased rate of infections, anastomotic leaks, reduced absorption of immunosuppressive medications, deficiency of folic acid, B12, iron, vitamin D, zinc, increased risk of hyperoxaluria, nephrolithiasis, and oxalate nephropathy [109,132,150]. The mortality rate associated with bariatric procedures was higher when operation was performed after KT rather than before KT: 3.5% versus 1% in the first 30 days and 7% versus 1% between 30 and 90 days [100]. While bariatric surgery achieves greater absolute weight loss, pharmacotherapy is often preferred over bariatric surgery post KT due to reduced perioperative risk, avoidance of medication absorption interference (critical for immunosuppressives), and lower incidence of surgical complications. Nevertheless, large-scale comparative data are lacking, and long-term effects on graft survival remain to be established.

The data concerning the safety and efficacy of SGLT2i in kidney transplant recipients comes from observational studies and one small RCT. There were no significant interactions with immunosuppressive drugs. There is more evidence to recommend the use of SGLT2i in patients with type 2 diabetes after KT, and no sufficient evidence in non-diabetic KT patients [151]. Retrospective analysis of KT patients with DM, treated with SGLT2i after kidney transplantation, showed improved glycemic control without safety concerns during follow-up of 3 years [152]. A systematic review and meta-analysis of DM patients using SGLT2i after KT summarized that these medications effectively lower HbA1C after 12 months of treatment and reduce body weight after 6 months of treatment without serious adverse events [153]. The comprehensive analysis of diabetic KT patients, which used SGLT2i within the first 3 months after kidney transplantation, showed significantly lower rates of all-cause mortality (adjusted hazard ratio 0.32), major adverse cardiac events (HR 0.48), and major adverse kidney events (HR 0.52) [154].

Besides all that was discussed about obesity and kidney transplantation, there are concerns that BMI alone do not represent a real problem concerning metabolically unhealthy obesity—sarcopenic obesity, cardiovascular kidney metabolic syndrome, and other associated co-morbidities [128].

Obesity increases complications after renal transplantation and can be a barrier to transplant access. Lifestyle modifications, dietary counseling, and increasing physical activity rarely lead to sustained weight loss. Bariatric surgery may help to overcome the barrier for kidney transplantation, and sleeve gastrectomy is associated with less postoperative complications. Pharmacotherapy for weight reduction before kidney transplantation is possible but has not been studied enough. Attempts at weight reduction should be continued after kidney transplantation, with pharmacotherapy being preferred over bariatric surgery.

Evidence on impact of obesity in kidney transplantation is summarized in Table 2.

## 5. Conclusions

Obesity is increasingly common among patients with CKD and ESKD, affecting disease progression, treatment eligibility, and clinical outcomes from childhood through adulthood. Its impact extends across both dialysis and kidney transplantation. This review synthesizes current evidence on underlying pathophysiology, alterations in body composition, dialysis-specific considerations, and transplant-related challenges, emphasizing the need for integrated and individualized management approaches.

Although a higher BMI has been associated with improved survival in dialysis patients—a phenomenon referred to as the “obesity paradox”—this apparent benefit does not mitigate the adverse cardiovascular, metabolic, and functional consequences of visceral adiposity and sarcopenic obesity. Therefore, comprehensive assessment beyond BMI and personalized management strategies are essential to optimize outcomes in this growing patient population.

Obesity also increases the risk of post-transplant complications and may restrict access to kidney transplantation. Lifestyle interventions alone often fail to achieve sustained weight loss. Bariatric surgery, particularly sleeve gastrectomy, has emerged as an effective option for weight reduction prior to transplantation, while pharmacological therapies show promise but remain insufficiently studied in this population. Importantly, weight management should continue after transplantation to improve long-term outcomes.

### 5.1. Key Messages for Clinical Practice

BMI alone is an inadequate measure of risk in ESKD; clinicians should incorporate body composition analysis (sarcopenia, visceral adiposity) and frailty assessment into routine evaluation.

In dialysis populations, apparent survival advantages associated with higher BMI (“obesity paradox”) should be interpreted in the context of muscle mass, nutritional reserve, and functional status.

For kidney transplant candidates, structured, individualized weight reduction—especially via GLP-1/GIP receptor agonists or laparoscopic sleeve gastrectomy—can improve listing eligibility while minimizing complications.

In transplant recipients, pharmacotherapy should generally be trialed before bariatric surgery due to lower perioperative risk and avoidance of immunosuppressive malabsorption, with surgery reserved for refractory, severe obesity.

### 5.2. The Most Important Knowledge Gaps in Evidence

Sparse pediatric-to-adult longitudinal studies on early-life obesity and lifetime kidney risk.

Absence of RCTs for obesity pharmacotherapies in ESKD and post-KT patients, limiting strong recommendations.

Unclear optimal timing/type of bariatric surgery relative to KT and lack of direct comparison with advanced medical therapies.

Insufficient multicenter data on robotic-assisted KT to validate its scalability for obese candidates.

Poorly understood effects of weight loss interventions on immunosuppressive drug levels post transplant.

### 5.3. Future Research Priorities

Long-term, pediatric-to-adult cohort studies elucidating the full impact of childhood obesity on renal structure, function, and progression to ESKD.

Implementation of standardized sarcopenia and visceral fat metrics in CKD clinical practice and research.

Large randomized controlled trials to evaluate pharmacotherapy for obesity in ESKD and post-KT populations, with endpoints including survival, graft outcomes, and cardiovascular risk.

Comparative studies of bariatric surgery versus advanced pharmacotherapy in post-KT patients.

## Figures and Tables

**Table 1:** Impact of obesity in dialysis patients.

Aspect	Key Findings	Citation
Prevalence in Dialysis	30–45% of HD patients are obese; rates higher in North America and Middle East; increasing trend in PD patients from 21.9% before 2000 to 47.3% in 2015.	Kramer 2006 [74]; Than 2020 [77]
Obesity Paradox in Dialysis	Higher BMI associated with improved short-term survival in HD and PD; each 1 kg/m^2^ ↑ * BMI → 3% ↓ ** all-cause mortality, 4% ↓ cardiovascular mortality; possible mechanisms include protein-energy reserves, cytokine sequestration, hemodynamic stability. However, long-term cardiovascular/metabolic risks remain high.	Johansen 2004 [108]; Ladhani 2017 [81]; Kalantar-Zadeh 2017 [83]; Park 2014 [84]
Body Composition Issues	Sarcopenic obesity common due to inflammation, metabolic acidosis, comorbidities; visceral fat associated with higher mortality despite BMI paradox. Waist circumference predictive of cardiovascular outcomes independent of BMI.	Azhar 2021 [79]; Oh 2017 [87]
HD-specific Challenges	Obesity complicates vascular access (AVF cannulation difficulty, deeper access); possible shorter access survival; challenges with central venous catheter placement; requires adaptations like superficialization/lipectomy.	Chan 2008 [91]; Okawa 2019 [92]; Naffouje 2016 [93]
PD-specific Challenges	↑ rates of catheter malfunction, exit-site infection, peritonitis, leaks, hernias; visceral fat can entrap catheter; icodextrin use mitigates weight gain; metabolic complications possible; PD not a contraindication but requires technical optimization.	Mehrotra 2009 [98]; Krezalek 2018 [99]; Obi 2018 [89]; Choi 2017 [103]
Dialysis Adequacy Issues	Kt/V calculations may underestimate dose for obese patients; requires longer HD sessions or extra PD exchanges; practicality sometimes limits adaptation.	Celebi-Onder 2012 [94]
Resource&Equipment Needs	Bariatric chairs, larger BP cuffs, extra-long PD catheters, specialized transfer equipment may be required in units.	Berger 2007 [96]
Complication Risks	Higher calciphylaxis prevalence—tensile stress from adipose tissue + arteriolar calcification; ↑ infection risk for large skin lesions.	Diwan 2020 [78]

* ↑—increase, ** ↓—decrease.

**Table 2:** Impact of obesity in kidney transplantation.

Aspect	Key Findings	Citation
BMI Thresholds for Listing	Many centers impose limits: 30–34 kg/m^2^: 2–24% centers; 35–39 kg/m^2^: 12–51% centers; >40 kg/m^2^: 6–62% centers; some exclude BMI > 40 outright. DESCARTES guidelines accept 30–34, KDIGO advises caution but not absolute exclusion.	Boerstra 2025 [120]; Pruthi 2018 [121]; Maggiore 2019 [122]; DESCARTES 2021 [123]; KDIGO 2020 [127]
Surgical/Post-Op Risks	↑ wound infection (RR = 3.13), wound dehiscence (RR = 4.85), lymphocele, delayed graft function (RR = 1.52), acute rejection (RR = 1.17), thrombotic events; prolonged hospital stay (+2.31 days), NODAT (RR = 2.24).	Lafranca 2015 [111]; Sood 2016 [113]
Graft and Patient Survival	Some meta-analyses show ↑ * mortality (RR = 1.52) in obesity; others show no difference post-2000; KT still offers survival benefit over dialysis regardless of BMI.	Lafranca 2015 [111]; Nicoletto2014 [115]; Krishnan 2015 [116]
Lifestyle Changes Pre-KT	Dietary counseling and physical activity efforts have limited long-term success in ESKD due to fatigue, anemia, and diet restrictions.	Lee 2021 [125]; Ikizler 2024 [126]; Drueke 2025 [128]
Pharmacotherapy Pre-KT	GLP-1RAs and dual GLP-1/GIP agonists show modest weight reduction (1–8 kg over 3–12 months) in small ESKD studies; limited evidence in advanced CKD; GI side effects common (up to 16%).	Wade 2025 [141]; Vanek 2024 [142]; Clemens 2023 [140]
Bariatric Surgery Pre-KT	SG and RYGB effective (up to ~80% excess weight loss, BMI drop 11 kg/m^2^); meta-analysis: 50% listed, ~30% transplanted post-surgery; SG preferred due to fewer complications, less malabsorption, no oxalate nephropathy risk.	Orandi 2020 [133]; Choudhury 2020 [134]; Di Napoli 2025 [135]; DESCARTES 2021 [123]
Robotic KT in Obese Patients	Robotic-assisted KT feasible in class III obesity with good graft/patient survival, minimal complications; can be combined with robotic SG for dual benefit.	Tzvetanov 2020 [137]; Spaggiari 2021 [138]
Post-KT Weight Gain	~20% prevalence of obesity post-KT within first year; driven by withdrawal of restrictions, increased appetite, inactivity, corticosteroids; GLP-1RA/tirzepatide promising, but untested in large KT RCTs.	Henggeler 2018 [144]; Mahzari 2024 [147]; Valencia-Morales2023 [149]; Frias 2021 [146]
Bariatric Surgery Post-KT	Effective for weight loss but ↑ surgery-related mortality (3.5% vs. 1% pre-KT), higher infection risk, delayed wound healing, drug malabsorption.	Modanlou 2009 [100]; Veroux 2021 [132]; Gazzetta 2017 [150]

* ↑—increase.

## Data Availability

No new data were created or analyzed in this study.

## Abbreviations

CKD	Chronic Kidney disease
ESKD	End-Stage Kidney Disease
BMI	Body Mass Index
HD	Hemodialysis
PD	Peritoneal Dialysis
ORG	Obesity-Related Glomerulopathy
TNFa	Tumor Necrosis Factor alpha
ROS	Reactive Oxygen Species
TREM2	Triggering Receptor Expressed on Myeloid Cells 2
RAAS	Renin–Angiotensin–Aldosteron System
ACE	Angiotensin-Converting Enzyme
GFR	Glomerular Filtration Rate
FF	Filtration Fraction
GH	Glomerular Hyperfiltration
FSGS	Focal Segmental Glomerulosclerosis
DM	Diabetes mellitus
PEW	Protein-Energy Wasting
AVF	Arteriovenous Fistula
AVG	Arteriovenous Graft
KDIGO	Kidney Disease: Improving Global Outcomes
KT	Kidney Transplant
LSG	Laparoscopic sleeve gastrectomy
RYGB	Rouxen Y gastric bypass
RAKT	Robotic-assisted kidney transplantation
GLP-1 RA	Glucagon-like peptide-1 receptor agonist
GIP RA	Glucose-dependent insulinotropic polypeptide receptor agonist
RSG	Robotic sleeve gastrectomy
RCT	Randomized clinical trials
